# Complete genome analysis of *Vibrio mimicus* strain SCCF01, a highly virulent isolate from the freshwater catfish

**DOI:** 10.1080/21505594.2019.1702797

**Published:** 2019-12-28

**Authors:** Zehui Yu, Erlong Wang, Yi Geng, Kaiyu Wang, Defang Chen, Xiaoli Huang, Ping Ouyang, Zhicai Zuo, Chao Huang, Jing Fang, Lizi Yin, Hongrui Guo, Zhijun Zhong

**Affiliations:** a College of Veterinary Medicine, Sichuan Agricultural University, Chengdu, Sichuan, P. R. China; b Laboratory Animal Center, Southwest Medical University, LuZhou, Sichuan, P. R. China; c College of Animal Science and Technology, Northwest A&F University, Yangling, Shaanxi, P.R. China; d College of Animal Science and Technology, Sichuan Agricultural University, Chengdu, Sichuan, P. R. China

**Keywords:** *Vibrio mimicus*, virulence factors, genome element, genomic comparison

## Abstract

*Vibrio mimicus* is a foodborne pathogen, which is widely distributed in the aquatic environment. Moreover, it is often involved in aquatic animal diseases. In recent years, *V. mimicus* is an emerging pathogen in some species of Siluriformes. The strain SCCF01 was isolated from yellow catfish (*Pelteobagrus fulvidraco*). In this study, we aimed to perform genomic analysis of *V. mimicus* strain SCCF01 to identify genetic features and evolutionary relationships. Information on gene function and classification was obtained by functional annotation, and circular graph of strain SCCF01 genome, which was created by Circos v0.64. Information on virulence genes (adhesion, flagellum system, exotoxin, and secretory system, etc.) was obtained by virulence genes annotation. Genome element prediction showed that most of the mobile elements were distributed in chromosome I. Therefore, chromosome I of SCCF01 genome has more plasticity than chromosome II and might be larger in size. Genomic linear relationship between the strain of *V. mimicu*s and strain SCCF01 was analyzed by linear pairwise comparison but was unable to determine the relationship. Gene family analysis predicted that the evolutionary direction of strain SCCF01 was: clinical strain → environmental strain → SCCF01 strain. Phylogenetic analysis showed that the strain SCCF01 was more closely related to environmental strains. According to gene family analysis and phylogenetic analysis, we speculated that strain SCCF01 has probably diverged from environmental strains.

## Introduction

*Vibrio mimicus* (*V. mimicus*) was initially considered as an atypical *V. cholerae,*^[1]^ which is closely related to *Vibrio cholerae. V. mimicus* is a widely distributed aquatic bacterium that can cause disease in humans and massive death of aquatic animals. It is a foodborne pathogen [2], which can cause gastroenteritis, diarrhea and food poisoning [3–5]. *V. mimicus* infecting was also common in aquaculture (can infect shrimp, crab, and fish) [6–11]. In recent years, *V. mimicus* is an emerging pathogen in some species of Siluriformes. The epidemiological features of *V. mimicus* showed short disease duration and high mortality rate, which eventually leads to substantial economic loss in Siluriformes farmhouses [9,10]. Since 2011, large-scale *V. mimicus* infectious outbreaks have occurred continuously in Siluriformes farms in China. *V. mimicus* strain SCCF01, which is a highly virulent strain isolated from yellow catfish (*Pelteobagrus fulvidraco*) in China, causes almost 100% mortality in yellow catfish [9,12].

At present, there are three complete genome sequences and eight draft genome sequences of *V. mimicus* available in Genbank genome database (five strains were isolated from human, five strains were isolated from the environment and only one strain SCCF01 was isolated from fish) (Supplementary Table S1). Genome sequence of strain SCCF01 was the only complete genome sequence of *V. mimicus* from infected aquatic animals. Previous studies have shown that *V. mimicus* strain SCCF01 natural infection can cause high mortality rate in fishes [9], and is also a highly virulent in the artificial infection experiment [12,13]. However, the genetic features and evolutionary strategies of *V. mimicus* from fish remain unknown.

## Materials and methods

### Sources of strain and genome sequences

*V. mimicus* strain SCCF01, which was isolated from diseased yellow catfish (*Pelteobagrus fulvidraco*) at a commercial aquaculture site in Southwest China [9]. Challenges showed that bath immersion of strain SCCF01 (10 [6] CFU·mL^−1^) caused 100% mortality of yellow catfish. The whole-genome sequence of *V. mimicus* was obtained by single-molecule real-time (SMRT) sequencing using platform PacBio RS II [12]. The complete genomic sequences of SCCF01 have been deposited in GenBank under the accession numbers CP016383 (chromosome I) and CP016384 (chromosome II). The other genomic sequences which were used for comparative genome analysis were downloaded from the National Center for Biotechnology Information (NCBI) (Supplementary Table S1).

### Analysis of genome plasticity

**General genomic features**: The genome of *V. mimicus* strain SCCF01 was annotated automatically using the GeneMarkS+ based on the NCBI Prokaryotic Genome Annotation Pipeline (PGAAP) [14]. **RNA prediction**: The rRNA identification was performed with RNAmmer 1.2 software [15], and the tRNA genes were predicted by tRNAscan-SE v2.0 [16]. **COG classification**: The predicted Open Reading Frame (ORF) sequences for the *V. mimicus* strain SCCF01 were translated into protein sequences and subsequently aligned against the COG database (http://www.ncbi.nlm.nih.gov/COG/). Accordingly, the predicted genes were divided into COG classes. Circular layouts were generated using Circos v0.64 (http://circos.ca/) [17]. **Virulence factor analysis**: Virulence factor database (VFDB) [18] was used to assess gene essentiality of ORFs predicted in SCCF01 with BLAST search. **Prediction of repeat elements**: Interspersed Repeat Sequences (IRS) and Tandem Repeat Sequences (TRS) in *V. mimicus* strain SCCF01 genome were screened by online program RepeatMasker 4.0.7 (http://www.repeatmasker.org/) using default parameters [19]. **Prediction of genomic islands**: IslandViewer 4 (http://www.pathogenomics.sfu.ca/islandviewer/browse/) [20] was applied to predict genomic islands in *V. mimicus* strain SCCF01 with default settings. **Prophages prediction**: Prophage sequences were predicted using Phage Search Tool (http://phast.wishartlab.com/) [21].

### Comparative genome analysis

**Collinearity analysis**: Global collinearity was identified using Mummer v3.23 by genome-wide sequence comparisons (http://mummer.sourceforge.net/) [22], and LASTZ v1.03.54 (http://www.bx.psu.edu) [23] provides genome-local sequence comparisons to determine the detailed collinearity (Translocation/Trans, Inversion/Inv and Trans+Inv) between two sequences. **Gene family analysis**: The pairwise alignments of the genome using BLAST [24] were performed to filter out untrustworthy results. Meanwhile, a gene family clustering table was constructed based on the results of alignment similarity by Hcluster-sg v 0.5.1 [25]. **Phylogenetic analysis**: The two phylogenetic gene trees (*Vibrio mimicus* species and *Vibrio* genus) were constructed based on locally collinear block searching in the sample and reference strains as previously reported [26]. The output file in HomBlocks alignments was input into RAxML [27] to construct the phylogenetic trees using the GTR+I + G model with a bootstrap value of 1,000. The phylogenetic trees were displayed and customized using Evolview (http://www.evolgenius.info/evolview/) [28]. **Structural variation (SV) identification**: The global alignments using Mummer v3.23 [22] were performed between SCCF01 and each reference strain, then Structural variation (SV) was identified using the LASTZ v1.03.54 [23] by pairwise alignment.

## Results and discussion

### General genomic features of V. mimicus strain SCCF01

**Figure 1.**
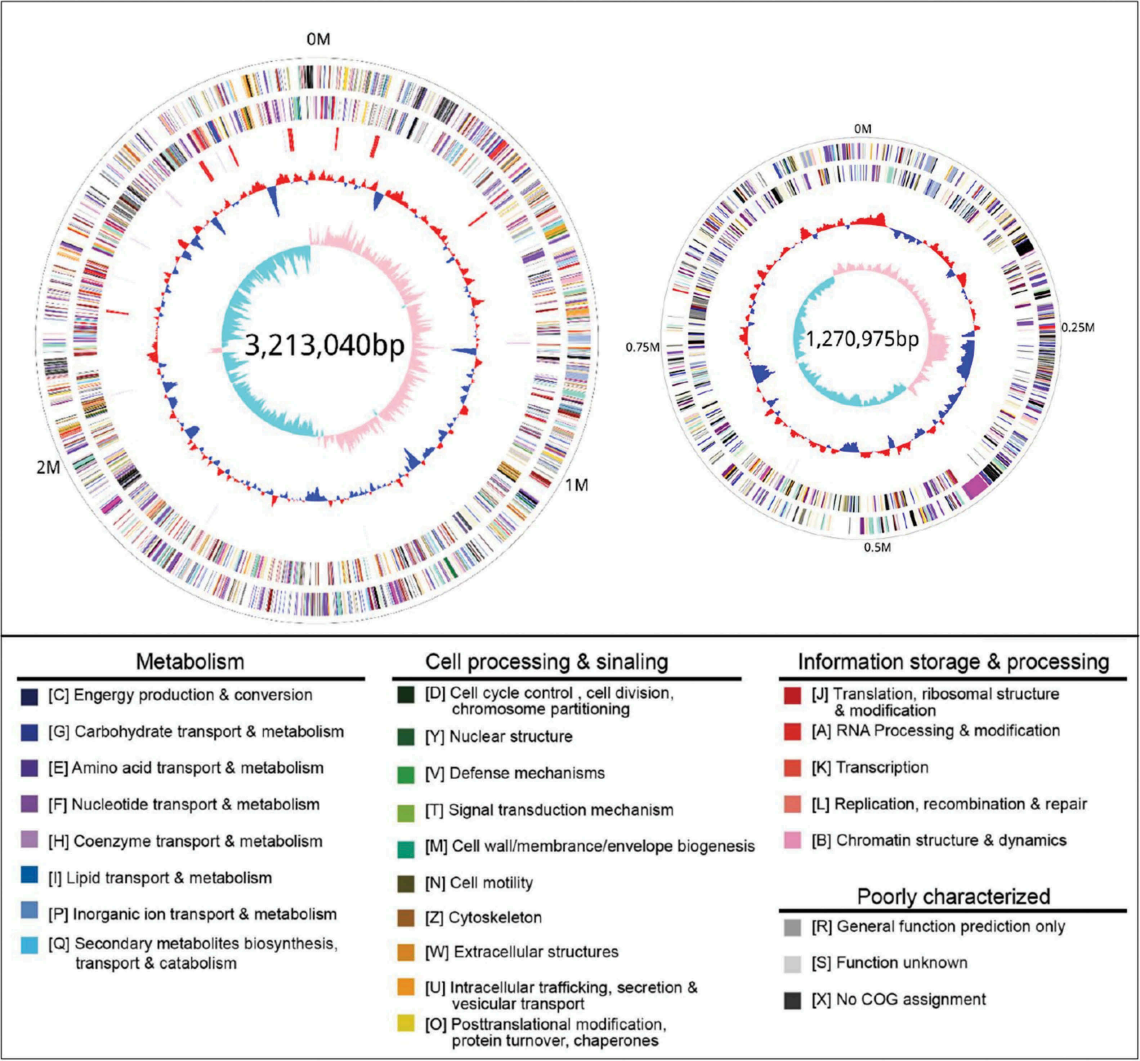
Circular graph of *V. mimicus* strain SCCF01 genome. Rings from the outermost to the center: (1) scale marks of the genome; (2) protein-coding genes on the forward strand; (3) protein-coding genes on the reverse strand; (4) tRNA (black) and rRNA (red) genes on the forward strand; (5) tRNA (black) and rRNA (red) genes on the reverse strand; (6) GC content; (7) GC skew. Protein-coding genes are color-coded according to their COG categories.

The genome of *V. mimicus* strain SCCF01 was sequenced using PacBio RS II with the P6-C4 Reagent Kit, which resulted in 35,089 pair polymerase reads with Read N50 of 12,135-bp and their characteristics are summarized in Table 1. After filtering, all reads were assembled into two circular chromosomes of 3,213,040 bp for Chromosome I and 1,272,975 bp for Chromosome II, with a G + C content of 46.61% and 45.88%, respectively (Supplementary Table S2). A total of 4,160 genes (4,018 CDSs and 140 RNA genes) were predicted in the SCCF01 genome by PGAAP (Table 1). The strain SCCF01 genome encodes 18 rRNAs and 69 tRNAs. The predicted ORFs are further classified into COGs functional groups (Figure 1) which summarized in Supplementary Table S3.

**Table 1.** The results of sequencing and assembly.

	Attribute	Value
PacBio statistic data	Polished Contigs	2
	Adapter Dimers (0–10bp)	0.0%
	Short Inserts (11-100bp)	0.0%
	Number of Bases	306,431,068
	Number of Reads	35,089
	N50 Read Length	12,135
	Mean Read Length	8,732
	Mean Read Score	0.84
	Mapped Reads	33,254
	Mapped Read Length of Insert	7,032
	Average Reference Length	1,500,987
	Average Reference Bases Called	100.0%
	Average Reference Consensus Concordance	100.0%
	Average Reference Coverage	56.73
Characteristics of the genome of *Vibrio mimicus* strain SCCF01 genome	genes (total)	4,160
	CDS	4,018
	RNA	142
	rRNAs	11, 10, 10 (5S, 16S, 23S)
	complete rRNAs	11, 10, 10 (5S, 16S, 23S)
	tRNAs	105
	ncRNAs	6

### Virulence factors

Based on the VFDB database [18], we scanned the *V. mimicus* SCCF01 genomes for virulence-related features. In total, we identified 107 putative orthologs involved in the production of any of the above virulence factors (summarized in Table 2). This reported dataset should be applied to the development of gene attenuated vaccine and can serve as the basis for future studies concerning interactions of *V. mimicus* strain SCCF01 and diseases. Here, we also compared the genomic virulence genes of *V. mimicus* SCCF01 with the clinical strain (ATCC33655) and environmental strain (ATCC33654). The three genomes analyzed shared 539 virulence genes in the core genome and the Specific virulence genes were 74 (SCCF01), 73 (ATCC33655) and 52 (ATCC33654) respectively (Supplementary Figure 2). Characteristics of common and specific virulence genes were summarized in Supplementary Table. The results showed that various virulence genes related to intestinal infections exists only in clinical strain (ATCC33655), such as cholera enterotoxin (ctx), accessory cholera enterotoxin (ace), zona occludens toxin(zot) and toxin co-regulated pilus (tcp). Interestingly, the other type VI secretion system (T6SS) gene cluster (VM_12775 ~ VM_12835) which is different with *Vibrio* exists in strain SCCF01. In addition, various virulence genes that are from other species exist only in strain SCCF01. Whether these extraneous virulence factors cause Siluriformes diseases need to be investigated further.

**Table 2.** Virulence factors of *V. mimicus* strain SCCF01.

	Annotation	Virulence factor	Virulence factor
Adherence	Accessory colonization factor	-	-
	Mannose-sensitive hemagglutinin	mshA~mshQ	VM13460 ~ VM13540
	Outer Membrane Protein U	OmpU	VM12035
	Toxin-coregulated pilus	-	-
	Type IV pilus	pilD, pilC, pilB, pilA	VM02850 ~ VM02865
Flagellum system	Capsular polysaccharide	flaA and flaC	VM03930 ~ VM03935
		flaE, flaD, flab, flaG	VM04235 ~ VM04250
		flhB	VM04355
		fliD, flaI, fliS, flrA, flrB, flrC	VM04255 ~ VM04280
		flgB ~ flgL	VM03875 ~ VM03925
		flgT, flgO, flgP, flgN, flgM, flgA	VM03835 ~ VM03860
	Flagella motor protein	motA and motB	VM10690 ~ VM10685
		motY and motX	VM10115, VM01930
	Flagella	flgA~flgN	VM19705 ~ VM19770
		fliE~fliR	VM04285 ~ VM04350
	Chemotaxis protein	cheY, cheZ, cheA, cheB, cheW	VM04615 ~ VM04645
		cheV and cheR	VM03865 ~ VM03870
Toxin	Hemolysin	VMH, TLH	VM17590, VM17595
	Enterotoxin	Enterotoxin	VM17705
	Proteases	Metalloproteases, Neuraminidase	VM20315, VM15685
Secretion system	type III secretion system	-	-
	EPS type II secretion system	epsC ~ epsN	VM00880 ~ VM00935
	type VI secretion system	vgrG-3, vasa ~ vasL, vipA~B	VM18140 ~ VM18220
Iron uptake	Heme receptors	HutA	VM16145
	Siderophore receptors	FhuA	VM14565
	Siderophores (Enterobactin, Vibriobactin and Aerobactin)	vctA, irgA	VM17540, VM17545
		viuA, viuB	-
		iutA	VM09630
	TonB1 system	ExbB1, ExbD1, TonB1	VM20125 ~ VM20135
	TonB2 system	ExbB2, ExbD2, TonB2	VM07220 ~ VM07230
	Transport of iron complexes	hmuV and hutC	VM08650 ~ VM08655
		FhuC, FhuD, FhuB	VM14550 ~ VM14560
		vctC, vctG, vctD, vctP	VM17550 ~ VM17565
	Feo system	FeoA and FeoB	VM04550 ~ VM04555
	Fbp system	FbpA, FbpB, FbpC	VM12160 ~ VM12150

### Genome element prediction

Mobile elements including repetitive elements, genomic islands, and phages within genomes have driven bacterial horizontal gene transfer and evolution [29–31].

Repetitive elements (also known as repeated sequences) are repetitive multiple copies of DNA sequences that do not have transcriptional activity. According to their structure, repetitive elements can be divided into Interspersed Repeat Sequences (IRS) and Tandem Repeats Sequence (TRS). Repetitive elements within genomes play an important role in the evolutionary process [32]. SCCF01 genome was screened by online program RepeatMasker, and the results (Table 3) showed that the total IRS percentage of the strain SCCF01 genome in Chromosome I and Chromosome II was 21.79% and 13.75%, respectively. Similarly, the total TRS percentage of the strain SCCF01 genome in Chromosome I and Chromosome II was 7.30% and 8.30%, respectively. The percentage of total IRS in Chromosome I was greater than Chromosome II. However, the percentage of total TRS in Chromosome II was greater than Chromosome I. The IRS is derived from transposable elements (TEs), that are largely responsible for horizontal gene transfer [33]. The TRS can exhibit high-mutation rates [34]. Therefore, we speculated that chromosome I is responsible for structural variation and chromosome II is responsible for single nucleotide change in the evolutionary process of strain SCCF01.

**Table 3.** Repeat elements prediction in *V. mimicus* strain SCCF01.

	Chromosome I			Chromosome II		
	number	length	percentage	number	length	percentage
SINE	26	1700 bp	5.29%	1	148 bp	1.16%
LINE	51	4310 bp	13.41%	15	1046 bp	8.23%
LTR	0	0 bp	0.00%	0	0 bp	0.00%
DNA element	14	991 bp	3.08%	7	553bp	4.35%
Unclassified	0	0 bp	0.00%	0	0 bp	0.00%
Total IRS	81	7001 bp	21.79%	23	1747 bp	13.75%
Satellites	0	0 bp	0.00%	0	0 bp	0.00%
Simple repeats	44	1870 bp	5.82%	16	836 bp	6.58%
Low complexity	9	475 bp	1.48%	5	218 bp	1.72%
Total TRS	53	0	7.30%	21	0	8.30%

**Figure 2.**
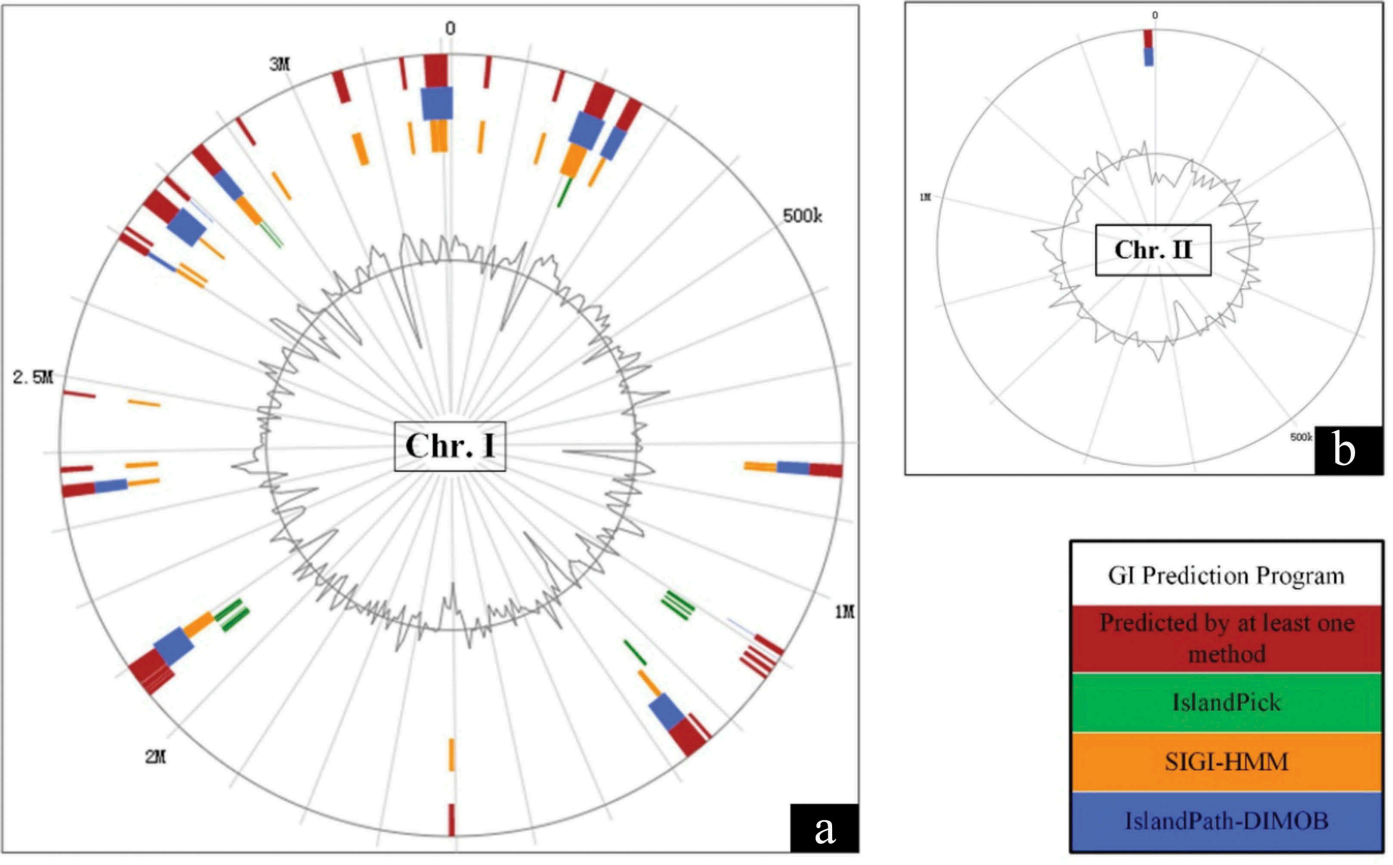
Genomic islands (GI) prediction in *V. mimicus* SCCF01 by IslandViewer; A: Chromosome I (Chr.I), Chromosome II (Chr.II).

Genomic islands (GI) are large genomic regions that mediated horizontal gene transfer in bacteria [31]. There are 16 GIs predicted in the genome of the strain SCCF01 by IslandViewer 4 and localization of predicted GIs as shown in Figure 2. Interestingly, 15 predicted GIs were located in the Chromosome I, in this case only one predicted GI was located in Chromosome II. The results of genomic islands prediction indicated that Chromosome I was more likely than Chromosome II to acquire genes via horizontal gene transfer.

**Figure 3.**
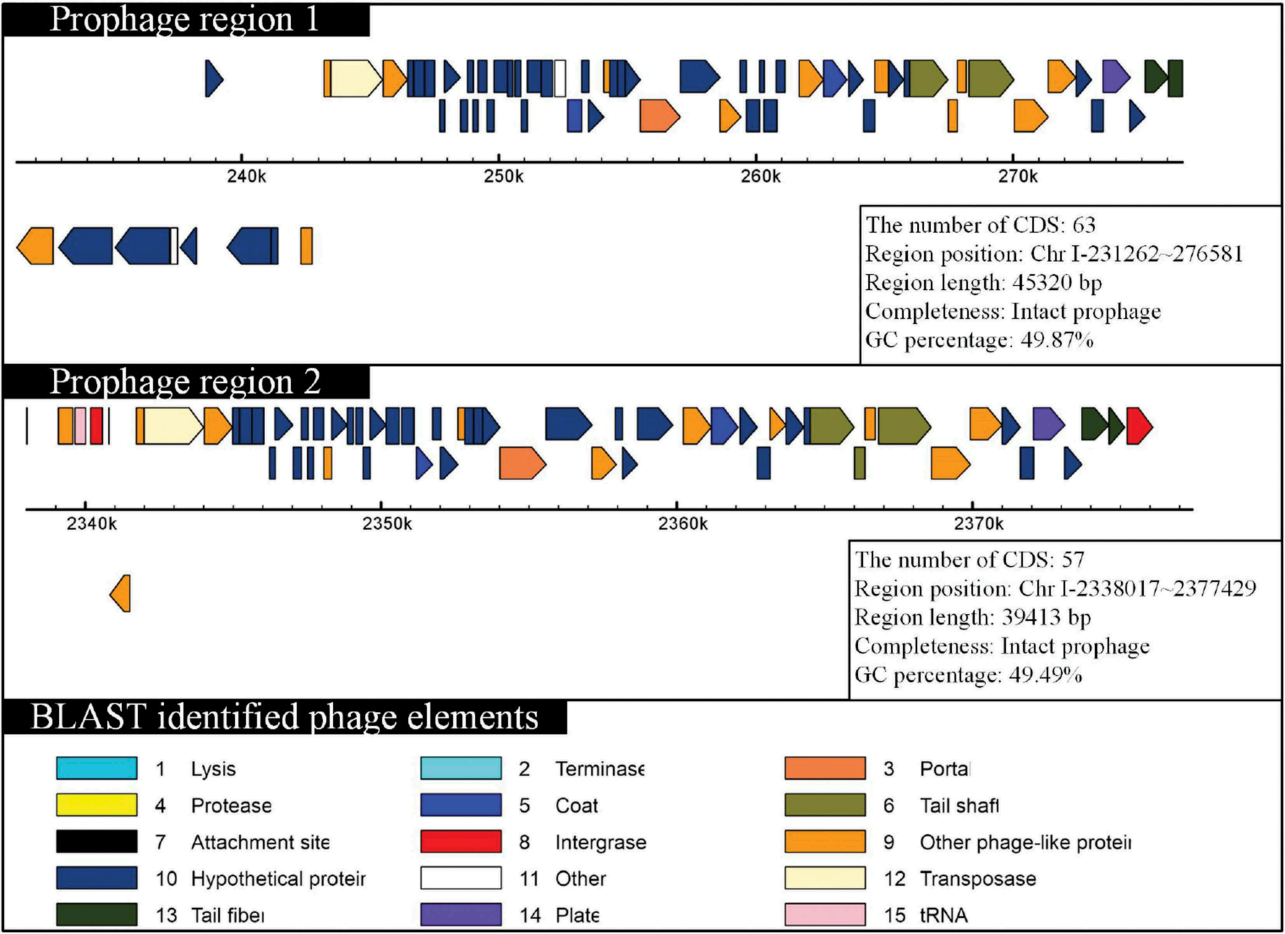
Schematic diagram of prophage organization.

Prophage sequences of 11 *V. mimicus* strains were predicted by PHAST and the features are listed in Supplementary Table S4. Prophages prediction showed that the strains (SCCF01, ATCC33654, ATCC33655, SX-4, and VM573) contained intact prophage sequences and other strains contained incomplete or questionable prophage sequences. *V. mimicus* strain SCCF01 uniquely harbored two integrated prophages in the large chromosome (chromosome I) of strain SCCF01, and its CDSs sharing greater identity to the *Vibrio* phage 12B12 [GenBank: NC_021070.1] (Figure 3). However, no prophage sequences could be detected in chromosome II of strain SCCF01. Recently, many researches have shown that prophage can mediate horizontal gene transfer [35,36]. The results of prophage prediction indicated that chromosome I was capable of horizontal gene transferring by a prophage.

In general, the majority of mobile elements (repetitive elements, genomic islands, and prophage) were detected in chromosome I. These mobile elements are connected closely to horizontal gene transfer contribution to acquire genes [33,35–37]. Therefore, chromosome I of the SCCF01 genome has more plasticity than chromosome II and chromosome I might be enlarged in size.

### Collinearity analysis

**Figure 4.**
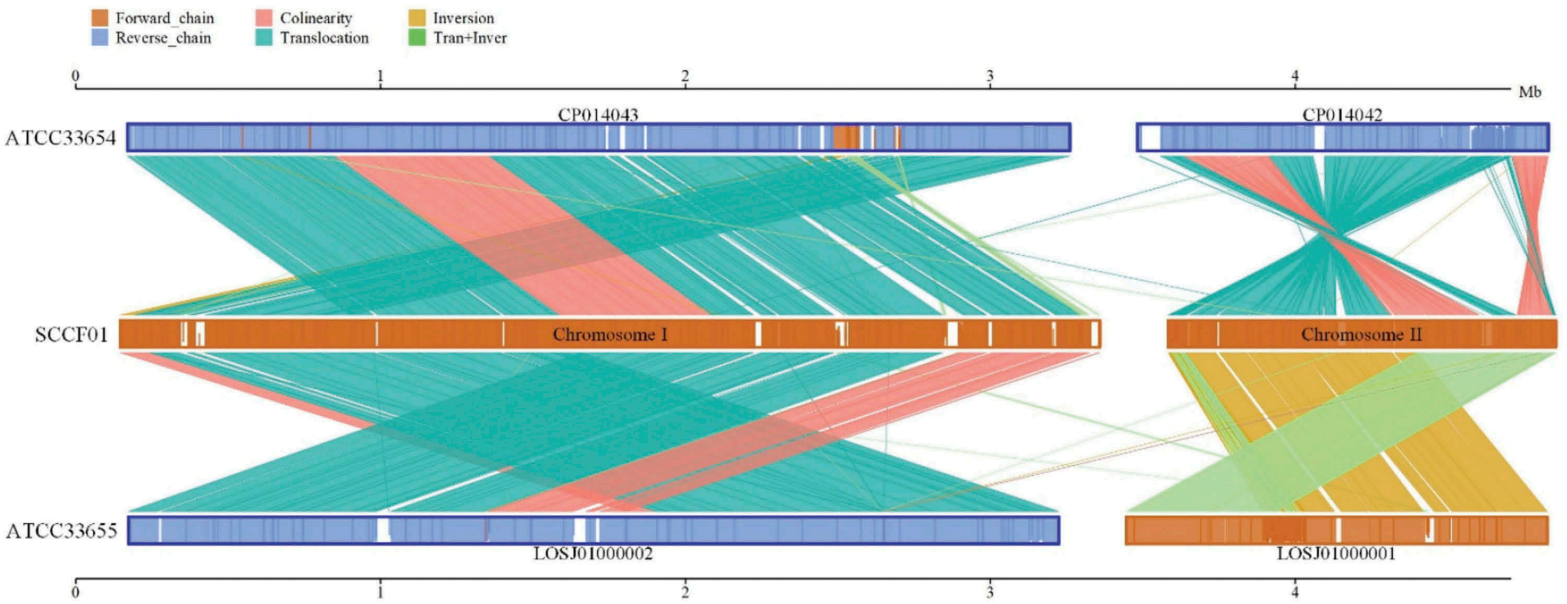
Linear pairwise comparison of the SCCF01 strain genome. Note: the upper axis and lower axis are standard strain genome, the medial axis is SCCF01 strain genome, sense strand is shown in the yellow box, antisense strand is shown in blue line, color depth in the box shows the similarity of alignment, full-fit indicates 100% similarity.

Herein, Mummer and LASTZ program were applied for a genome-wide collinearity analysis between the SCCF01 genome and standard strains (ATCC33654 and ATCC33655). Our analysis of the *V. mimicus* SCCF01 genome, suggested that the evolution of the strain SCCF01 genome structure is marked by interchromosomal rearrangements (Figure 4). Structural variation (translocation, inversion, deletion, insertion, and complexindel) is shown in Supplementary Figure 1. Genome-wide collinearity relation and detailed structural variation can be visualized. However, it is difficult to confirm evolutionary relationships.

### Gene family analysis

A gene family is a set of several similar genes, formed by duplication of a single original gene. The statistics of gene family numbers were obtained according to the cluster of orthologous group based on protein sequences of SCCF01, ATCC33654, ATCC33655, VM223, and MB-451. Gene family analysis showed that the number (genes number, genes in families, unclustered genes, family number, and unique families) of strain SCCF01 were calculated more than other strains (Table 4). In addition, the number (genes number, genes in families, unclustered genes, family number, and unique families) of environmental strains (ATCC33654 and VM223) were computed more than clinical strain (ATCC33655 and MB-451). The gene gain/loss events might have occurred during the evolution of the genus *Vibrio*, and the gene gain events in the evolutionary process of *V. mimicus* and *V. cholerae* were more frequent than the gene loss events [38]. Therefore, gene family analysis predicted that the evolutionary direction of strain SCCF01 was clinical strain → environmental strain → SCCF01 strain.

**Table 4.** The result of gene family analysis.

Strain	SCCF01	ATCC33654	ATCC33655	VM223	MB-451
Genes number	4,031	3,874	3,791	3,902	3,841
Genes in families	3,786	3,638	3,608	3,698	3,674
Unclustered genes	245	236	183	204	167
Family number	2,846	2,748	2,787	2,823	2,821
Unique families	46	19	13	24	6

Note (From left to right): 1. Genes number: Total number of genes, 2. Genes in families:Total number of genes in families, 3. Unclustered genes: The number of, 4. Family number: The gene family numbers, 5. Unique families: The unique numbers.

### Phylogenetic analysis

**Figure 5.**
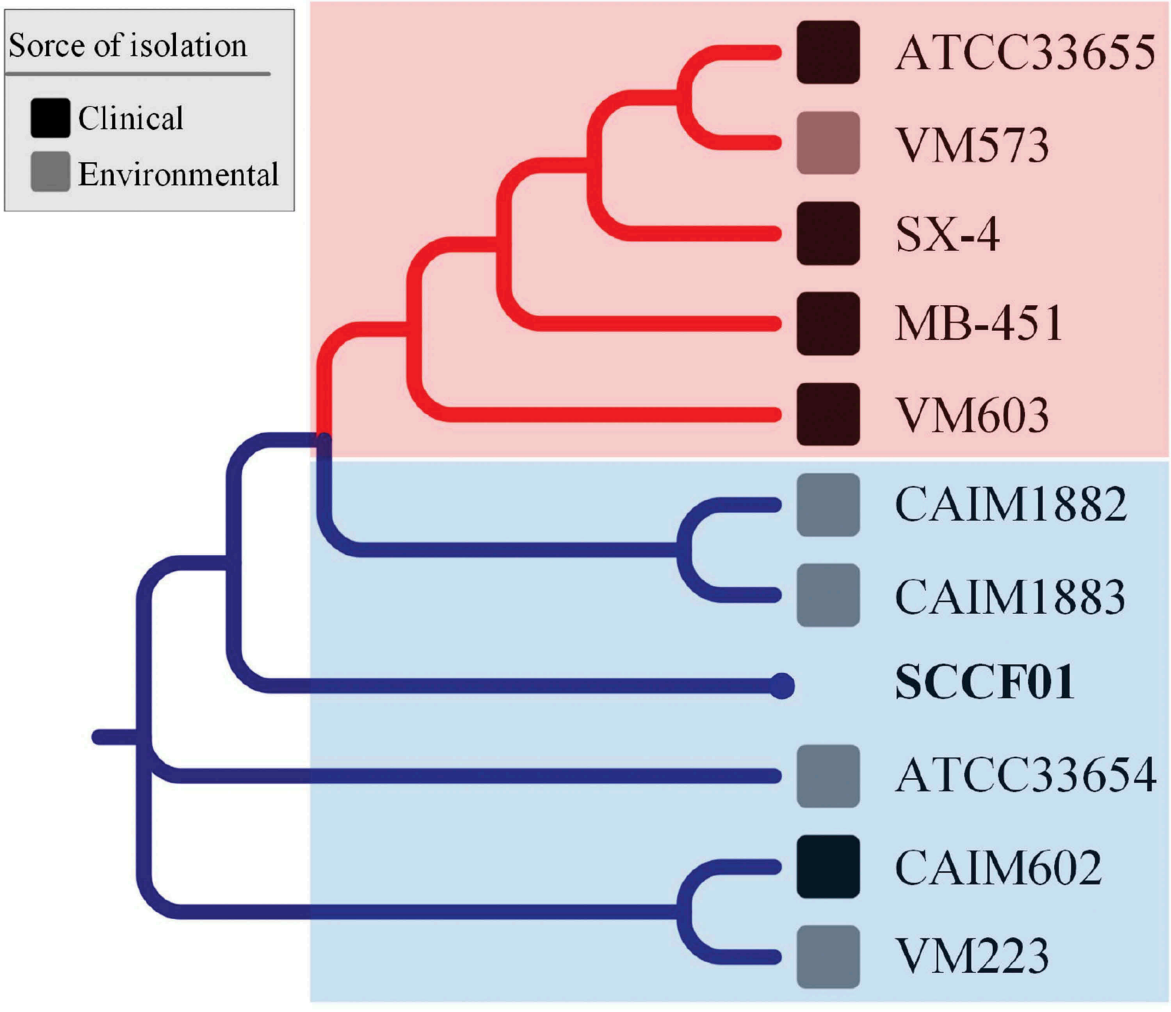
Phylogenetic tree of *Vibrio mimicus* based on locally collinear block searching.

In order to determine the phylogenetic relationship of the SCCF01, genome tree analysis was performed from *V. mimicus* species (Figure 5) and *Vibrio* genus (Supplementary Figure 2) based on locally collinear block searching. The phylogenetic tree from *V. mimicus* species (Figure 5) showed that the isolates were roughly divided into two clusters: clinical *V. mimicus* (ATCC33655, SX-4, and MB-451) and environmental *V. mimicus* (CAIM1882, CAIM1883, ATCC33654, SCCF01, and VM223). The evolutionary relationships inferred by this tree suggest that SCCF01 is more closely related to the environmental isolate.

The phylogenetic tree from *Vibrio* genus (Supplementary Figure 3) showed that the strain SCCF01 was classified into the *V. mimicus* cluster and further proved that the strain SCCF01 was determined to be *V. mimicus* on the genome level.

## Conclusions

First, Genome analysis of *V. mimicus* strain SCCF01 revealed common basic features. The information of virulence (adhesion, flagellum system, exotoxin, and secretory system) was obtained by virulence genes annotation and will be useful for the development of gene attenuated vaccine and pathogenesis study for this pathogen. Secondly, chromosome I of the SCCF01 genome has more plasticity than chromosome II and might be larger in size. Finally, we speculate that the strain SCCF01 has probably diverged from environmental strains based on gene family analysis and phylogenetic analysis.
