# Supplementary material for: Complete genome analysis of Vibrio mimicus strain SCCF01, a highly virulent isolate from the freshwater catfish
Source: Virulence. 2019 Dec 28;11(1):23–31. doi: 10.1080/21505594.2019.1702797 (PMC6961728; doi:10.1080/21505594.2019.1702797)
Supplement: Supplemental Material [file kvir-11-01-1702797-s001.docx]

**Supplementary Figure 1.** Graph visualization of SV detection for SCCF01 strain**.**


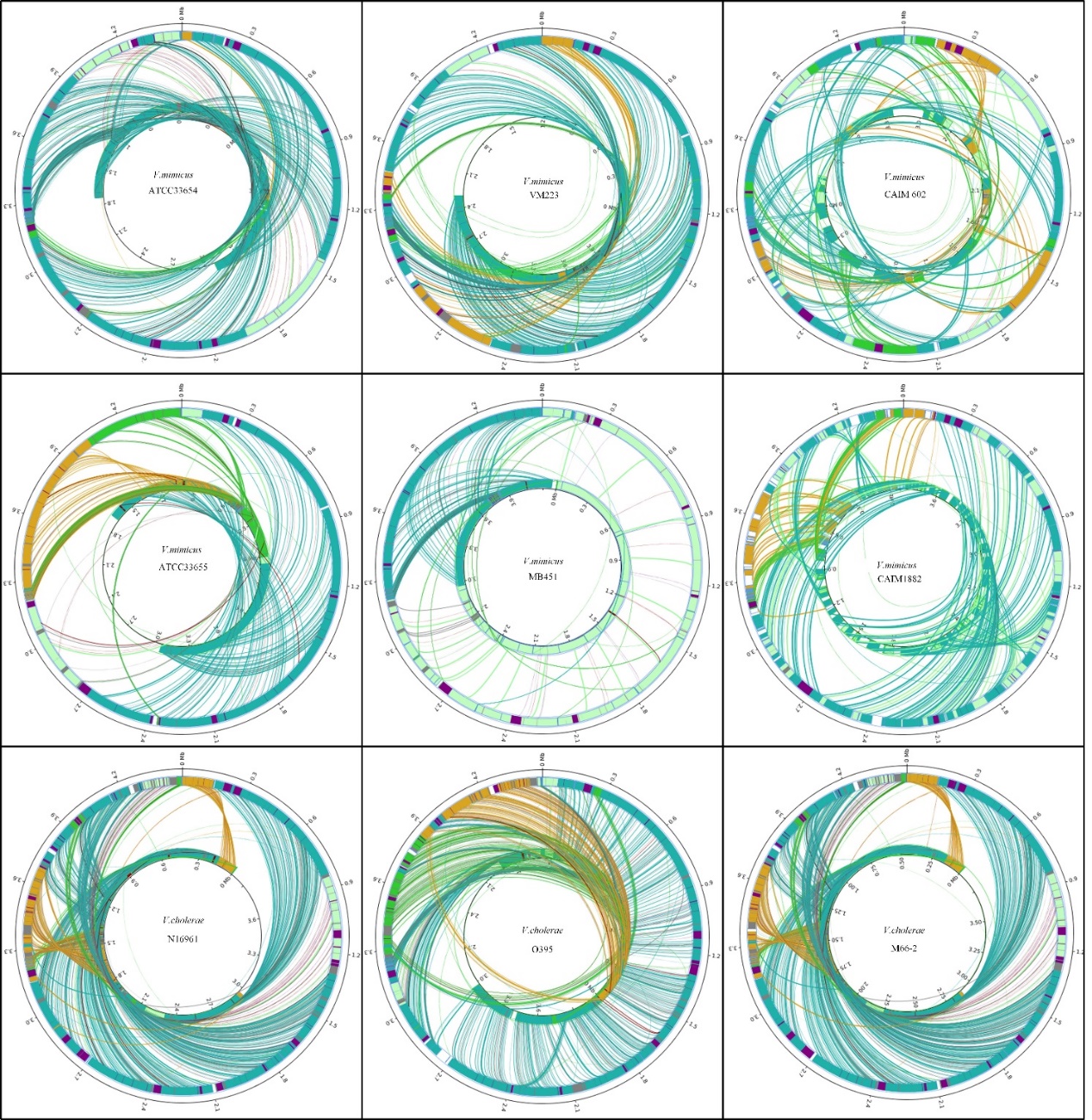


**Supplementary Figure 2:** Venn Diagram showing the distribution of virulence genes in the genomes of SCCF01, clinical strain (ATCC33655) and environmental strain (ATCC33654).


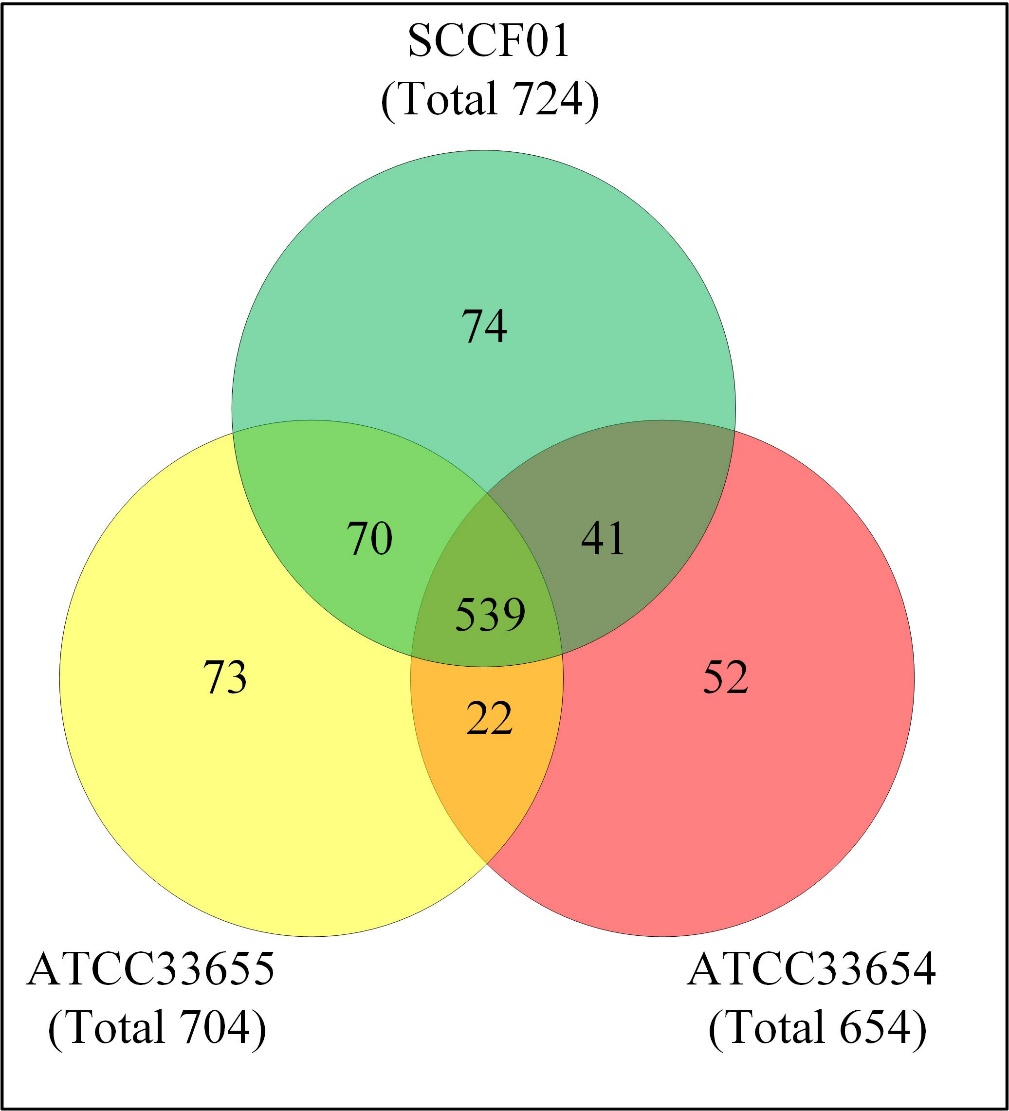


**Supplementary Figure 3:** Phylogenetic tree of usual vibrio base on genome**.**

**
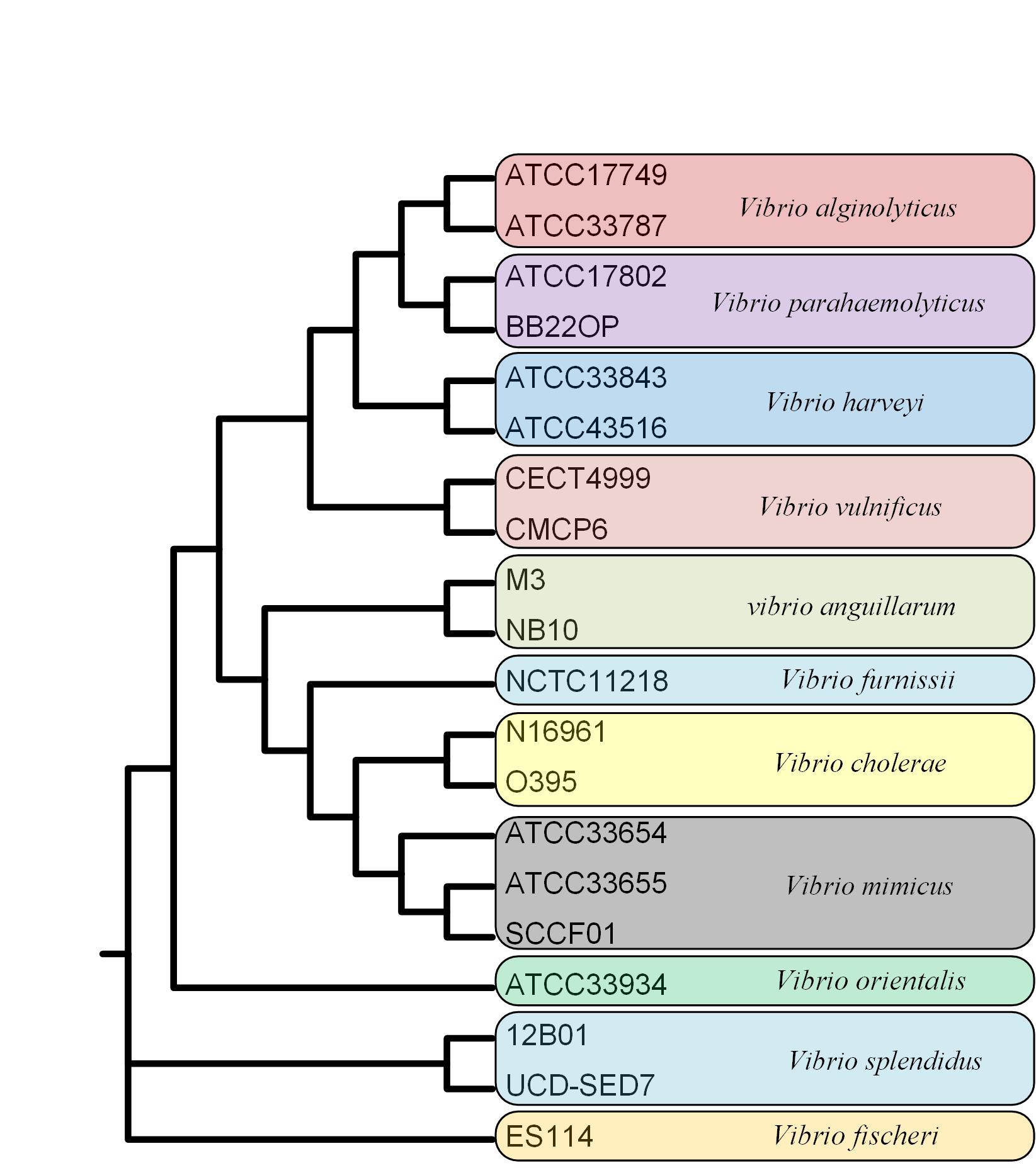
**

**Supplementary Table 1:** The information of genome used in comparative genome analysis**.**

| **Strain** | **Source of isolation** | **Accession no.** | **Technology** | **Reference** |
| --- | --- | --- | --- | --- |
| *v.mimicus* ATCC33654 | Lake water | CP014042.2, CP014043.2 | Pacbio+ Illumina | NCBI |
| *v.mimicus* ATCC33655 | Human | LOSJ00000000.2 | Pacbio+ Illumina | NCBI |
| *v.mimicus* VM223 | Bivalve in Saõ Paulo, Brazil | ADAJ00000000.1 | Sanger+454 | (Hasan et al., 2010) |
| *v.mimicus* MB-451 | Patient from Matlab, Bangladesh | ADAF00000000.1 | Sanger+454 | (Hasan et al., 2010) |
| *v.mimicus* CAIM 602 | Patient from North Carolina | AOMO00000000.1 | IonTorrent | (Guardiola-Avila et al., 2013) |
| *v.mimicus* CAIM 1882 | Water in Guaymas, Sonora, Mexico | AWWY00000000.1 | IonTorrent | (Guardiolaavila et al., 2016) |
| *v.mimicus* CAIM 1883 | Water in Guaymas, Sonora, Mexico | AWWL00000000.1 | IonTorrent | (Guardiolaavila et al., 2016) |
| *v.mimicus* SX-4 | Diarrhea patient form Shanxi, China | ADOO00000000.1 | Solexa | (Wang et al., 2011) |
| *v.mimicus* VM603 | Patient with gastrointestinal infection | ACYU00000000.1 | 454 | (Thompson et al., 2009) |
| *v.mimicus* VM573 | Riverine water in the Brazilian | ACYV00000000.1 | 454 | (Thompson et al., 2009) |

**References**

Guardiola-Avila, I., Acedo-Felix, E., Noriega-Orozco, L., Yepiz-Plascencia, G., Sifuentes-Romero, I., Gomez-Gil, B., 2013. Draft Genome Sequence of Vibrio mimicus Strain CAIM 602T. Genome Announc 1, e0008413.

Guardiolaavila, I., Acedofelix, E., Sifuentesromero, I., Yepizplascencia, G., Gomezgil, B., Noriegaorozco, L., 2016. Molecular and Genomic Characterization of Vibrio mimicus Isolated from a Frozen Shrimp Processing Facility in Mexico. Plos One 11, e0144885.

Hasan, N.A., Grim, C.J., Haley, B.J., Chun, J., Alam, M., Taviani, E., Hoq, M., Munk, A.C., Saunders, E., Brettin, T.S., 2010. Comparative genomics of clinical and environmental Vibrio mimicus. Proceedings of the National Academy of Sciences of the United States of America 107, 21134-21139.

Thompson, C.C., Vicente, A.C.P., Souza, R.C., Vasconcelos, A.T.R., Vesth, T., Alves, N., Ussery, D.W., Iida, T., Thompson, F.L., 2009. Genome taxonomy of Vibrios. 9, 258.

Wang, D., Wang, H., Zhou, Y., Zhang, Q., Zhang, F., Du, P., Wang, S., Chen, C., Kan, B., 2011. Genome Sequencing Reveals Unique Mutations in Characteristic Metabolic Pathways and the Transfer of Virulence Genes between V. mimicus and V. cholerae. Plos One 6, e21299.

**Supplementary Table 2:** Results of de novo assembly**.**

|  | **Chromosome I** | **Chromosome II** |
| --- | --- | --- |
| Genbank accession | CP016383 | CP016384 |
| Genome size | 3,213,040 bp | 1,270,975 bp |
| GC-content | 46.61% | 45.88% |
| Integrity | Closed | Closed |
| Coding region | 88.0% | 87.0% |
| Mean gene length | 979.3 bp | 966.1 bp |
| gene density | 0.898 genes per kb | 0.900 genes per kb |

**Supplementary Table 3:** The functional annotation in COG database**.**

| **Category (number/Ratio)** | **COG symbol** | **Functional description** | **Number** | **Ratio(%)** |
| --- | --- | --- | --- | --- |
| Cellular processes and signaling  (961/26.03%) | D | Cell cycle control, cell division, chromosome partitioning | 35 | 0.95% |
|  | M | Cell wall/membrane/envelope biogenesis | 174 | 4.71% |
|  | N | Cell motility | 161 | 4.36% |
|  | O | Posttranslational modification, protein turnover, chaperones | 145 | 3.93% |
|  | T | Signal transduction mechanisms | 286 | 7.75% |
|  | U | Intracellular trafficking, secretion, and vesicular transport | 114 | 3.09% |
|  | V | Defense mechanisms | 46 | 1.25% |
|  | W | Extracellular structures | 0 | 0.00% |
|  | Y | Nuclear structure | 0 | 0.00% |
|  | Z | Cytoskeleton | 0 | 0.00% |
| Information storage and processing (646/17.50%) | A | RNA processing and modification | 1 | 0.03% |
|  | B | Chromatin structure and dynamics | 1 | 0.03% |
|  | J | Translation, ribosomal structure and biogenesis | 192 | 5.20% |
|  | K | Transcription | 285 | 7.72% |
|  | L | Replication, recombination and repair | 167 | 4.52% |
| Metabolism  (1331/36.06%) | C | Energy production and conversion | 184 | 4.99% |
|  | E | Amino acid transport and metabolism | 314 | 8.51% |
|  | F | Nucleotide transport and metabolism | 81 | 2.19% |
|  | G | Carbohydrate transport and metabolism | 258 | 6.99% |
|  | H | Coenzyme transport and metabolism | 146 | 3.96% |
|  | I | Lipid transport and metabolism | 82 | 2.22% |
|  | P | Inorganic ion transport and metabolism | 200 | 5.42% |
|  | Q | Secondary metabolites biosynthesis, transport and catabolism | 66 | 1.79% |
| Poorly characterized  (753/20.40%) | R | General function prediction only | 434 | 11.76% |
|  | S | Function unknown | 319 | 8.64% |

**Supplementary Table 4: Statistics in** Prophages prediction.

| **Strain** | **Number** | **Length** | **Completeness** | **CDS** | **Region position** | **Possible phage** |
| --- | --- | --- | --- | --- | --- | --- |
| SCCF01 | Region 1 | 45.3Kb | intact | 63 | ChrI (231262-276581) | Vibrio 12B12 |
|  | Region 2 | 39.4Kb | intact | 57 | ChrI (2338017-2377429) | Vibrio 12B12 |
| ATCC33654 | Region 1 | 13.8Kb | incomplete | 8 | ChrI (1475670-1489535) | Salmon BP12C |
|  | Region 2 | 20.9Kb | incomplete | 23 | ChrII (32855-53756) | Entero 933W |
|  | Region 3 | 31.5Kb | intact | 41 | ChrII (583194-614755) | Vibrio K139 |
| ATCC33655 | Region 1 | 9.8Kb | incomplete | 7 | ChrII (1206351-1216225) | Bacill Nemo |
|  | Region 2 | 21Kb | intact | 30 | ChrII (682950-703992) | Vibrio CTX |
|  | Region 3 | 49.4Kb | intact | 49 | ChrII (963451-1012880) | Pseudo NP1 |
| VM-223 | Region 1 | 30.4Kb | incomplete | 9 | 666690-697143 | Salisa 1 |
|  | Region 2 | 9.8Kb | incomplete | 7 | 4204749-4214623 | Bacill Nemo |
| MB451 | Region 1 | 14.6Kb | questionable | 16 | 4275596-4290221 | Salmonella phage 19 |
| CAIM 602 | Region 1 | 23Kb | incomplete | 26 | 2516898-2539900 | Entero 933W |
| CAIM 1882 | Region 1 | 19Kb | incomplete | 15 | 3739301-3758341 | Stx2 vB EcoP |
| CAIM 1883 | Region 1 | 22.5Kb | incomplete | 20 | 3122060-3144621 | Entero 933W |
|  | Region 2 | 20.3Kb | incomplete | 14 | 3744818-3765168 | Stx2 vB EcoP 24B |
| SX-4 | Region 1 | 33.8Kb | intact | 53 | 875812-909616 | Vibrio 12B12 |
|  | Region 2 | 11.3Kb | incomplete | 11 | 1542105-1553502 | Escher D108 |
| VM603 | Region 1 | 9.8Kb | incomplete | 7 | 2525063-2534937 | Bacill Nemo |
| VM573 | Region 1 | 33.4Kb | intact | 45 | 424344-457792 | Pseudo NP1 |
|  | Region 2 | 10.4Kb | intact | 12 | 4109484-4119898 | Vibrio CTX |
